# Top Selling (2026) Small Molecule Orphan Drugs: A Journey into Their Chemistry

**DOI:** 10.3390/ijms24020930

**Published:** 2023-01-04

**Authors:** Davide Benedetto Tiz, Luana Bagnoli, Ornelio Rosati, Francesca Marini, Luca Sancineto, Claudio Santi

**Affiliations:** Group of Catalysis, Synthesis and Organic Green Chemistry, Department of Pharmaceutical Sciences, University of Perugia, Via del Liceo 1, 06123 Perugia, Italy

**Keywords:** FDA, EMA, orphan drugs, synthesis, new therapies

## Abstract

This review describes, from a chemical point of view, the top “blockbuster” small molecule orphan drugs according to their forecasted sales in 2026. Orphan drugs are intended for the treatment, prevention, or diagnosis of a rare disease or condition. These molecules are mostly addressed to the treatment of rare forms of cancer. The respiratory and central nervous systems represent other common therapeutic subcategories. This work will show how the orphan drugs market has significantly grown and will account for a consistent part of prescriptions by 2026.

## 1. Introduction

Orphan drugs, by definition, are intended for the treatment, prevention, or diagnosis of a rare disease (RD) or condition [1]. For the Food and Drug Administration (FDA), “supporting the development and evaluation of new treatments for rare diseases is a key priority” [2].

According to the European Medicine Agency (EMA), Between 5000 and 8000 distinct rare diseases exist [3]. In the early 1980s, rare diseases accounted for 5000 types [4].

The Greek-derived term “orphan disease” is used to designate diseases that affect only small numbers of individuals [5]. To date, there is no unique and clear definition of an orphan disease. In the USA, it is defined as one that affects fewer than 200,000 individuals; in Japan, the number of patients having such a disease is 50,000, while Australia sets its limit at 200 [6].

The European Union and the USA have implemented legislation to stimulate the development of drugs for patients suffering from rare diseases. The European Parliament with the Council of 16 December 1999 on Orphan Medicinal Products [7] and the American Orphan Drug Act (1983) [8], followed by the Orphan Drug Regulation (1993) [9], are clear examples of such legislation.

Companies and other drug developers can request an orphan drug designation, and, in the case of the USA, the FDA will grant such a designation if the drug meets specific criteria [10]. Among the benefits of an orphan drug designation are the eligibility for a federal research grant, a grant of 7-year market exclusivity, and up to a 50% tax credit (until 2018) for clinical trials that meet the requirements [2]. From 2018 onwards, the tax credit was reduced from 50% to 25% by the Trump Administration [11]. Having a significant medical benefit is one of the key criteria for the application of the regulation.

## 2. Top Selling Orphan Drugs (Forecast 2026)

The development and evaluation of new treatments for rare diseases are one of the priorities of pharmaceutical agencies (FDA/EMA). They can grant an orphan drug designation to a drug or biological product to prevent, diagnose, or treat a rare disease or condition. This designation guarantees certain advantages both in terms of tax breaks and for periods of market exclusivity after approval. The majority of orphan diseases possess genetic origins and are life-threatening and/or chronically debilitating [12]. Most new drug modalities first make their mark in rare diseases, as these conditions are often genetically defined.

In addition to their genetic roots, orphan diseases can be caused by helminth, protozoan, and bacterial infections or even by environmental poisoning [13]. 

From an ethical point of view, the orphan drug concept is intended to help patients suffering from rare conditions. From an economic point of view, pharmaceutical companies can benefit, given the above-mentioned incentives and relaxed regulations. 

Novel drug discovery approaches, from gene editing to AI-powered screening and drug design, will play an important role in the development of new orphan drugs [14]. 

Orphan drug sales growth significantly outpaces that of the wider pharmaceutical market, and Big Pharma’s fortunes are ever more closely linked to orphan drugs.

In support of this evidence, it has to be mentioned that sales have increased about 10% per year between 2005 and 2011 [13], and it is estimated that each of the top 10 “blockbuster” orphans in 2026 will be worth between $3 billion and $13 billion [14].

**Figure 1:**
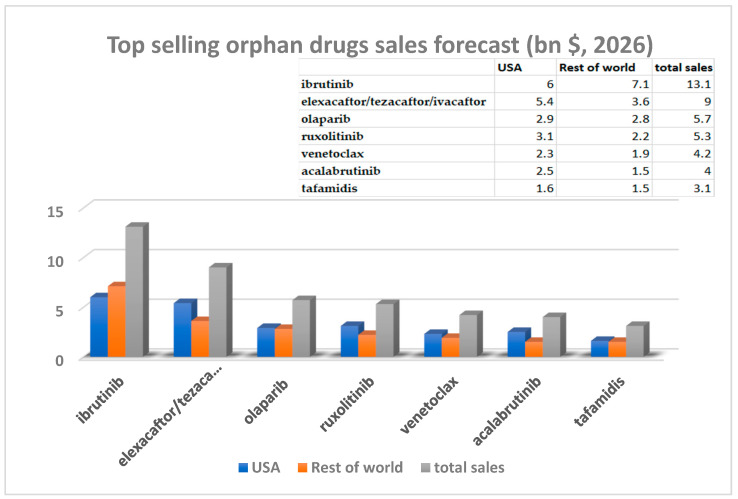
Top selling orphan drugs sales (bn $, forecasted sales for 2026) [14].

In 2026, among the top-selling orphan drugs (small molecules) are ibrutinib (brand name Imbruvica^®^), elexacaftor/tezacaftor/ivacaftor (brand name Trikafta^®^), olaparib (brand name Lynparza^®^), ruxolitinib (brand name Jakafi^®^), venetoclax (brand name Venclexta^®^), acalabrutinib (brand name Calquence^®^), and tafamidis (brand name Vyndaqel^®^) [14] (Figure 1).

The forecast for €mbruvica^®^ is outstanding, not only from the sales point of view but also because the European market will surpass the traditionally dominant USA market.

The goal of this review is to shed more light on the chemistry and sales of orphan blockbusters. Synthetic preparation and therapeutic use of each of these drugs will be discussed.

### 2.1. Ibrutinib (Brand Name Imbruvica^®^)

Bruton tyrosine kinase (BTK) signaling plays a fundamental role in B-cell development and in immunoglobulin synthesis. Ibrutinib (**10**) is an orally bioavailable BTK inhibitor and irreversibly binds to BTK at the Cysteine-481 residue [14]. It exhibits a low nanomolar potency (0.5 nM) against BTK [15]. Ibrutinib has been approved by the FDA for the treatment of several tumors, such as mantle cell lymphoma, chronic lymphocytic leukemia (CLL), Waldenstrom’s macroglobulinemia, marginal zone lymphoma, and chronic graft-versus-host disease in allogeneic stem cell transplantation [16,17].

Its acrylamide moiety reacts with C481 to form a covalent bond in the BTK kinase domain [18]. Ibrutinib was discovered at Celera Genomics. In 2006, Pharmacyclics (today part of AbbVie) initiated the preclinical and clinical development of ibrutinib. In December 2011, Pharmacyclics and the Janssen division of Johnson & Johnson entered into an agreement to jointly develop and market ibrutinib [19].

The synthesis of ibrutinib will b” discussed herein. Two main routes [19], small-laboratory scale and industrial, will be detailed. 

**Scheme 1:**
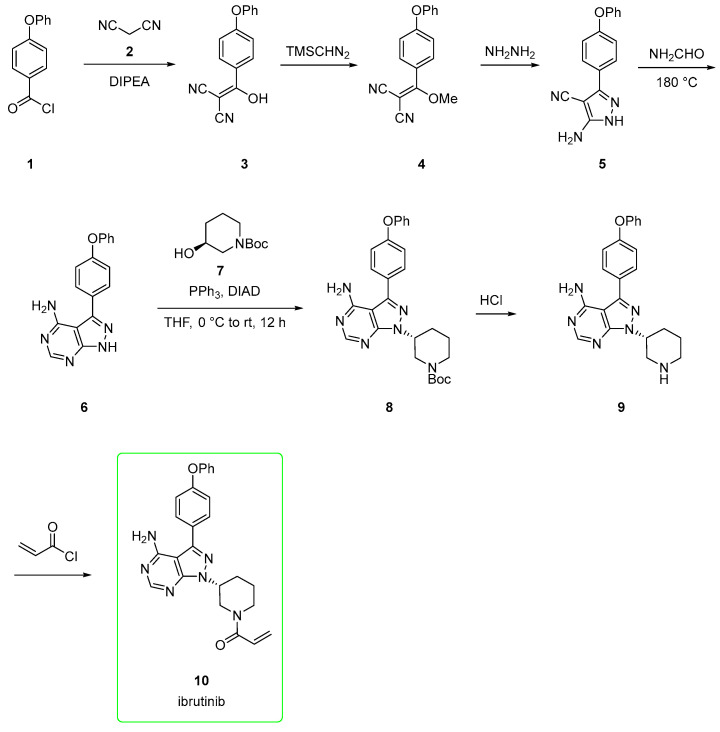
Medicinal chemistry route to ibrutinib (**10**) [20,21,22,23,24,25].

The small-scale route [20,21,22,23,24,25] starts from 4-phenoxybenzoyl chloride (**1**), which is reacted with malononitrile **2** to afford alkenol **3**. This is methylated upon the addition of trimethylsilyldiazomethane to yield derivative **4**. The addition of hydrazine to **4** yielded the amino-pyrazole **5**, which is, in turn, converted to pyrazolo-pyrimidine **6** via adding formamide at 180 °C. The subsequent Mitsunobu reaction between **6** and (*S*)-*tert*-butyl 3-hydroxypiperidine-1-carboxylate **7** afforded derivate **8** with a corresponding inversion of configuration at the alcoholic carbon center. The BOC-removal from **8** gave the free piperidine derivative 9. This was acylated with acryloyl chloride to provide ibrutinib (**10**, Scheme 1). This pathway allowed chemists to explore different heterocycle via Mitsunobu and different substituents at the free *N*-piperidine moiety.

**Scheme 2:**
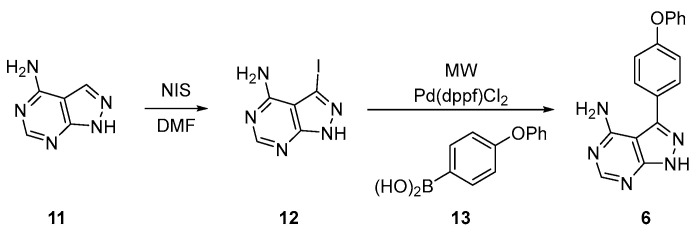
Alternative synthesis for synthon **6**.

An alternative pathway (Scheme 2) with fewer steps involves the preparation of synthon **6** in only two steps. The initial 1*H*-pyrazolo[3,4-*d*]pyrimidin-4-amine **11** is iodinated using *N*-iodosuccinimide (NIS) in DMF to afford **12**. This was subjected to microwave-assisted Suzuki-Miyaura coupling involving boronic acid **13** and [1,1′-Bis(diphenylphosphino)ferrocene]dichloropalladium(II), Pd(dppf)Cl_2_] as a catalyst to afford **6**. This is then subjected to the Mitsunobu procedure, as seen in Scheme 1.

The industrial route [26,27] is differentiated from the academic by avoiding the use of non-green Mitsunobu chemistry and expensive (*S*)-*tert*-butyl 3-hydroxypiperidine-1-carboxylate.

Moreover, it avoids the use of hydrazine hydrate, a suspected carcinogen that decomposes at higher temperatures.

**Scheme 3:**
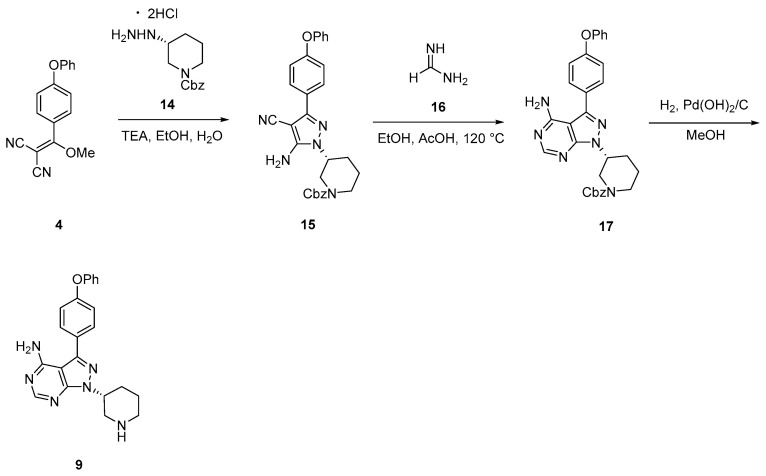
Industrial route [26,27] to intermediate **9** used in the preparation of ibrutinib.

The synthesis (Scheme 3) starts from the dinitrile **4**, which is transformed into pyrazole **15** via adding CBZ-protected€)-hydrazine **14**. Then, **15** was transformed into pyrazolo-pyrimidine **17** by mixing it with formamidine **16** at 120 °C. The subsequent removal of the CBZz-protecting group afforded **9** in good yield and purity after recrystallization in methanol (80% yield and 92.5% purity). Finally, **9** was used for subsequent acylation.

The forecasted sales for ibrutinib for 2026 are stunning. It is not, therefore, surprising that alternative synthetic pathways have appeared. Among them, and important to be mentioned, is the convergent Mitsunobu/displacement route [28,29,30]. In this route, a Mitsunobu reaction is performed at an earlier stage than the installation of the diphenyl ether group.

### 2.2. Elexacaftor/Tezacaftor/Ivacaftor (Brand Name Trikafta^®^)

Launched by Vertex Pharmaceuticals [14], elexacaftor (**31**)-tezacaftor(**48**)-ivacaftor(**62**) is a newly approved triple-combination cystic fibrosis transmembrane conductance regulator (CFTR) [31]. Cystic fibrosis (CF) is the most common, life-limiting autosomal recessive disease in Caucasians and is caused by defects in the production of the CFTR ion channel [32]. The CFTR is a tunnel-shaped chloride channel responsible for controlling the transport of anions and water in and out of epithelial cells [33].

Small-molecule modulators that directly interact with CFTR can aid in protein folding (“correctors”) and/or increasing channel function (“potentiators”) [34]. A recent study in 2022 has shown that when used alone, elexacaftor partially corrected interdomain assembly defects in phenylalanine deleted (Δ508) CFTR, but when combined with a type I corrector (e.g., tezacaftor), it did so fully [35].

#### 2.2.1. Elexacaftor

**Scheme 4:**
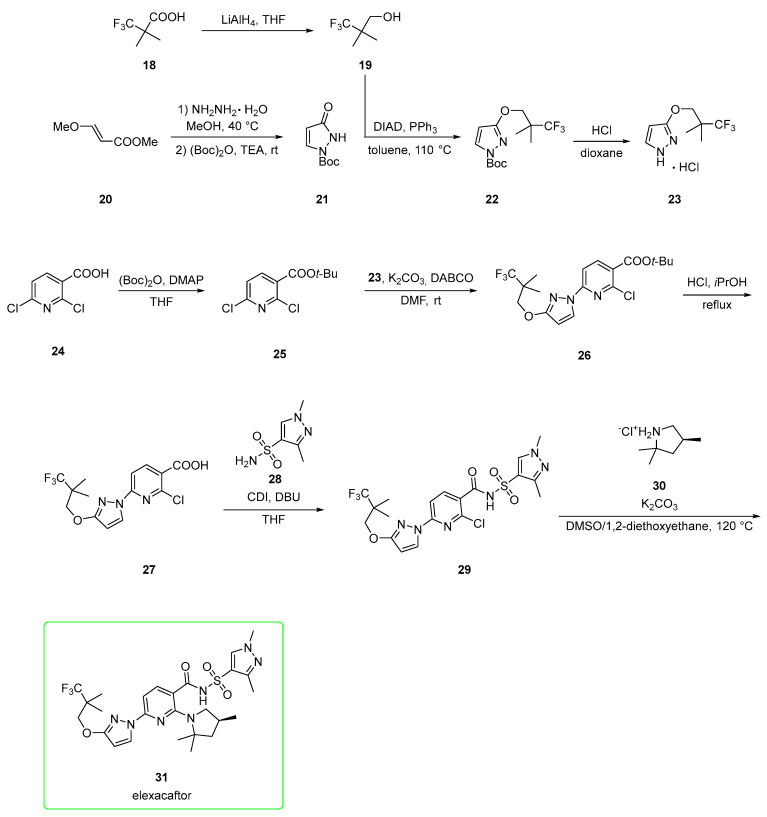
Preparation of elexacaftor (**31**) [31,34,36].

Elexacaftor has been described as both a corrector and potentiator of CFTR [36]. A first synthetic pathway (Scheme 4) was elaborated by Vertex [31,34,36]. It developed from the reduction of 3,3,3-trifluoro-2,2-dimethylpropanoic acid **18** to alcohol **19**, which is used in the Mitsunobu condensation of pyrazolone **21**, which is prepared from met€ (*E*)-3-methoxyacrylate **20** upon the addition of hydrazine hydrate and subsequent BOC protection. Mitsunobu product **22** was subjected to BOC deprotection with HCl to afford hydrochloric salt **23**.

Intermediate **23** was used under alkaline conditions in the nucleophilic addition to *t*-Bu ester **25** (prepared from 2,6-dichloropyridine-3-carboxylic acid **24**) to yield **26**. Deprotection of *t*-Bu ester **26** by using HCl at reflux generated free carboxylic acid **27**. This was activated by 1,1′-carbonyldiimidazole (CDI) and coupled with sulfonamide **28** to afford acyl sulfonamide **29**, which was used in the last step, the nucleophilic addition of pyrrolidine salt **30** in the presence of potassium carbonate to produce elexacaftor (**31**). The authors claim that the overall yield for the seven convergent steps was 29%, with the most problematic step being the Mitsunobu condensation (57%).

**Scheme 5:**
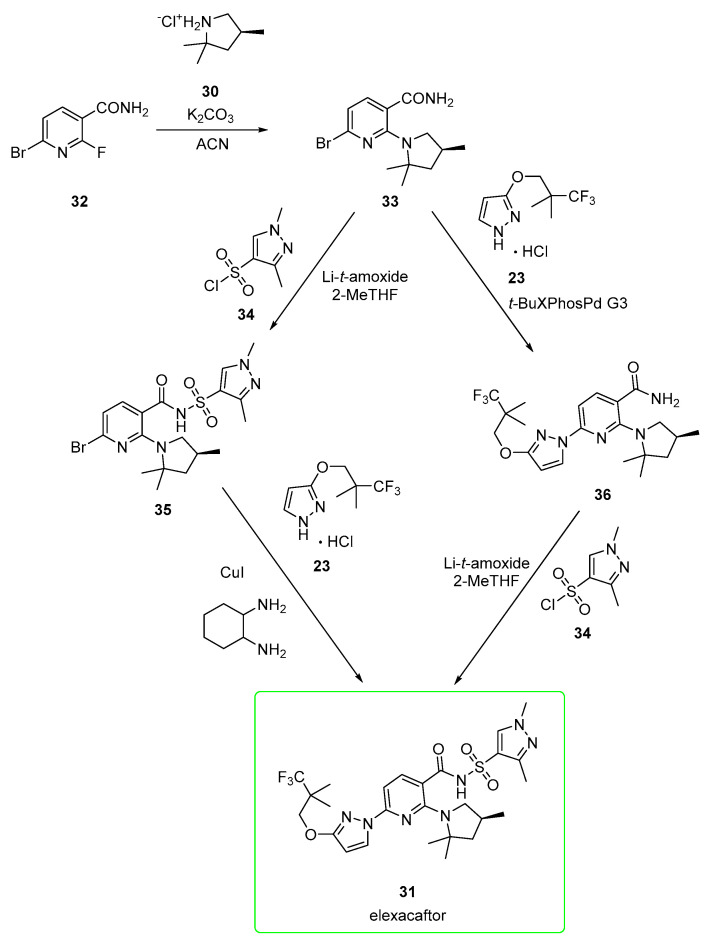
Alternative, more expeditious preparation [37] of elexacaftor (**31**).

An alternative pathway [37] (Scheme 5), again patented by Vertex, employed fewer steps. The starting material is a halogenated nicotinamide derivative **32**.

S_N_Ar reaction of **32** with pyrrolidine **30** in acetonitrile and K_2_CO_3_ afforded amide **33**, which was made nucleophilic by treatment with lithium *tert*-amoxide in 2-MeTHF as a solvent and treated with sulfonyl chloride **34** to yield acyl sulfonamide **35**. The eventual copper-catalyzed reaction between **35** and **23** afforded elexacaftor **31**. Alternatively, synthon **33** was first converted into pyrazole derivate **36** in a Buchwald–Hartwig amination employing third generation palladacycle catalyst (tBuXPhos Pd G3). Derivate **36** was lastly converted into elexacaftor **31** via the same conditions (lithium *tert*-amoxide in 2-MeTHF as a solvent, treated with sulfonyl chloride **34**) used to synthesize intermediate **35**.

#### 2.2.2. Tezacaftor

Tezacaftor, is a CFTR corrector [32]. Its function is to correct the positioning of the CFTR protein on the cell surface to permit proper channel formation and an improved flow of water and salts across the cell membrane [38]. Tezacaftor is approximately 99% bound to plasma proteins, mainly albumin [39].

**Scheme 6:**
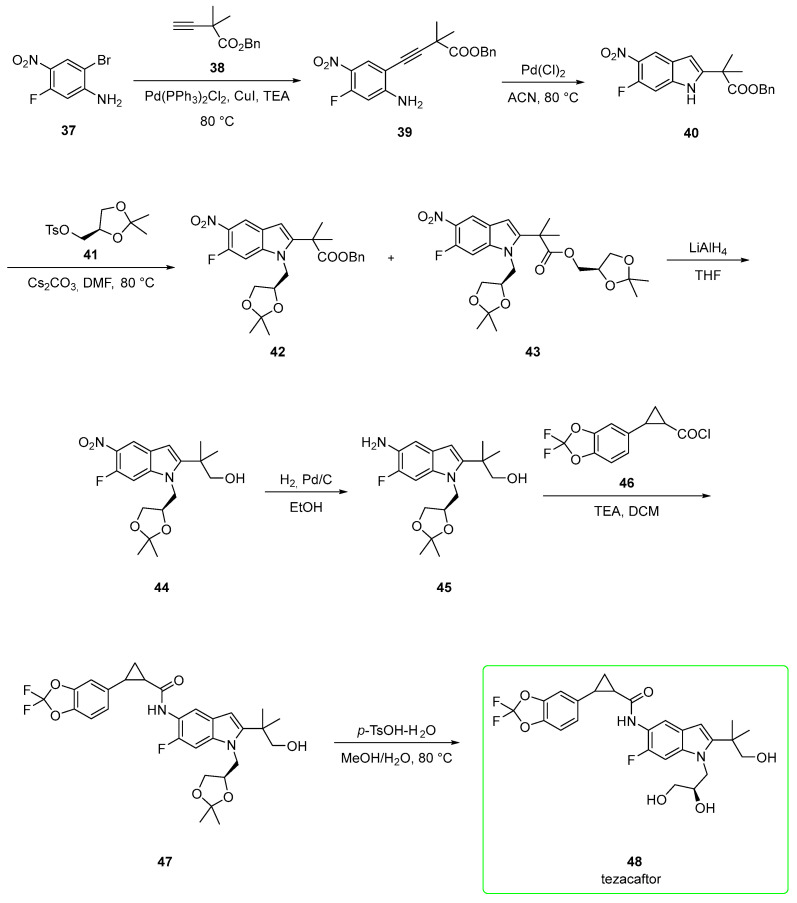
Preparation of tezacaftor (**48**) [33,40,41].

Its synthesis (Scheme 6) [33,40,41] starts from 2-bromo-5-fluoro-4-nitroaniline **37**. Sonogashira coupling [*Bis*(triphenylphosphine)palladium(II) dichloride] between **37** and alkyne **38** gave disubstituted alkyne **39**, which was subjected to cyclization under Pd(Cl)_2_ conditions to afford indole **40**. The indolic *N*H was alkylated with tosylate **41** in the presence of cesium carbonate to yield **42** and the undesired **43** (the transesterification product). Compound **42** was treated with LiAlH_4_ to yield the corresponding primary alcohol **44**. This underwent nitro reduction to yield indolamine **45**. Then, the nucleophilic attack of **45** to acyl chloride **46** in the presence of triethylamine afforded **47**. In the last step, the removal of the acetal protecting group afforded tezacaftor **48** in a moderate yield (47%) after chromatography.

**Scheme 7:**
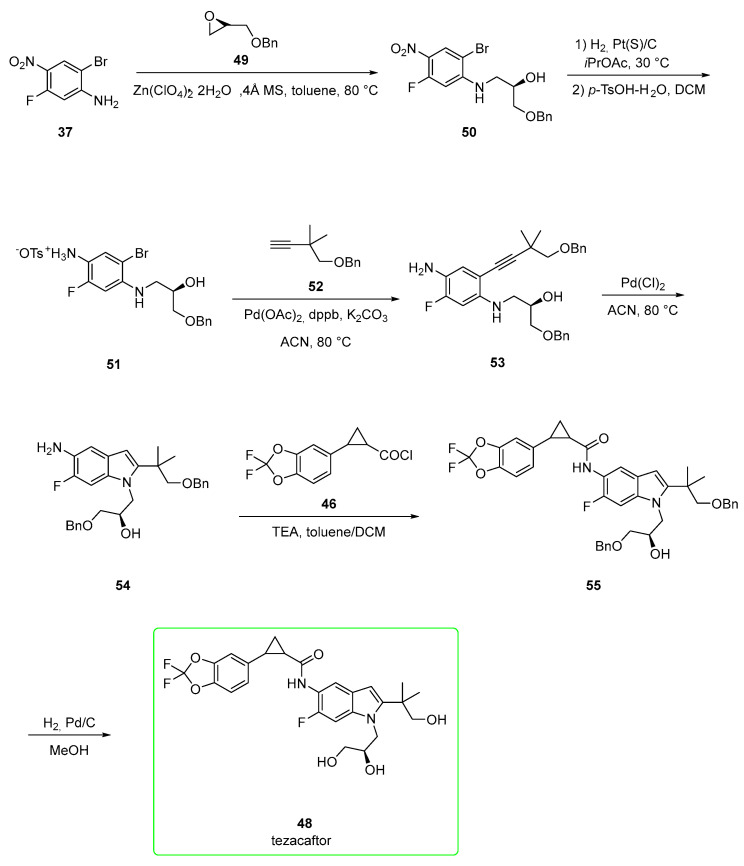
Alternative route to tezacaftor (**48**) [32,41].

An alternative pathway for tezacaftor (Scheme 7) involves the initial opening of (*S*)-epoxide **49** by 2-bromo-5-fluoro-4-nitroaniline (**37**) catalyzed by zinc perchlorate and 4 Å molecular sieves to afford intermediate **50**. Catalytic hydrogenation (platinum catalyst) of the nitro group followed by treatment with *p*-toluenesulfonic acid generated the anilinium salt **51**. The corresponding free base of **51** was subjected to Sonogashira coupling in copper-free conditions with alkyne **52** to yield disubstituted alkyne **53**. Indole formation catalyzed by Pd(Cl)_2_ in acetonitrile afforded intermediate **54**. The aromatic amine group of **54** was coupled with acyl chloride **46** in the presence of triethylamine to yield cyclopropyl amide **55**. Benzyl groups of **55** were removed under a hydrogen atmosphere (H_2_, Pd/C)) to generate tezacaftor (**48**). Yield for the last two steps ranged from 68% to 84% after crystallization from 2-PrOH/heptane [42]. This pathway affords the final product in better yields than those previously shown in Scheme 6.

#### 2.2.3. Ivacaftor

Ivacaftor (**62**) is a potentiator (it enhances the chloride transport) of the cystic fibrosis transmembrane conductance regulator (CFTR), and it is the first drug to be licensed for use that treats an underlying cause of cystic fibrosis [43]. Similar to tezacaftor, ivacaftor is also metabolized extensively in humans. In vitro and in vivo data indicate that ivacaftor is metabolized primarily by CYP3A4 and CYP3A5 [39,44].

**Scheme 8:**
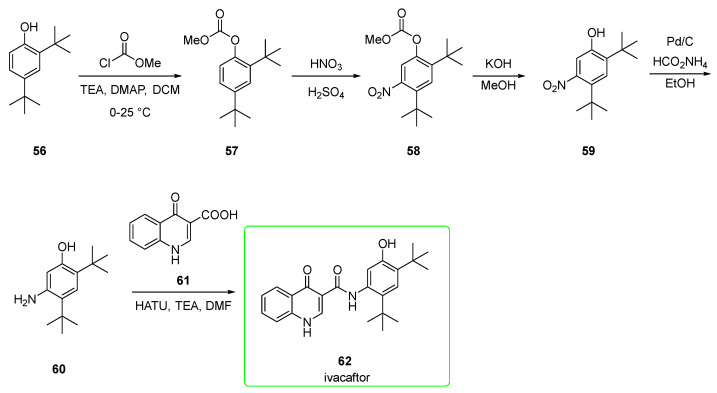
Preparation of ivacaftor (**62**) [45].

Its synthesis (Scheme 8) was first reported by Vertex [45]. The initial 2,4-di-*tert*-butylphenol (**56**) was protected with methyl chloroformate at its phenolic function to yield methyl carbonate **57**. The nitration afforded mainly derivate **58** (the undesired 6-nitro regioisomer is obtained in a ratio of 1:8). Hydrolysis of **58** provided phenol **59**, which underwent reduction of the nitro group under Pd/ammonium formate conditions to yield precursor **60**. This was eventually transformed into ivacaftor (**62**) via condensation with quinoline **61** upon HATU/TEA activation.

Protection of the phenol as the electron-withdrawing carbonate likely also minimizes the ortho/para-directing effect of the oxygen substituent, allowing nitration to occur primarily ortho/para to the *tert*-butyl groups [32].

**Scheme 9:**
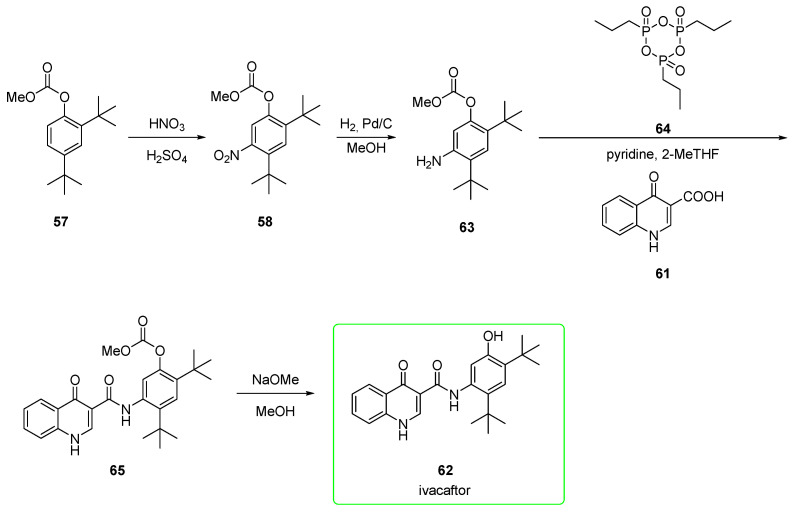
Alternative preparation of ivacaftor (**62**) [46].

An alternative pathway (Scheme 9) [46] encompasses similar steps but in a different order. In particular, the hydrolysis of the carbonate was carried out as the final step. Nitro compound **58** was reduced to aniline **63**. This was condensed with quinoline **61** upon activation of the carboxylic acid with propanephosphonic acid anhydride **64** to yield precursor **65**. This was eventually transformed after alkaline hydrolysis into ivacaftor **62**. No yields were provided for these reported steps. The protection of the phenol as the electron-withdrawing carbonate is likely to minimize its ortho/para-directing effect, as seen in the previous synthesis.

### 2.3. Olaparib

Olaparib (**72**, brand name Lynparza^®^) was approved by the FDA for the first-line maintenance treatment of breast cancer susceptibility gene (BRCA)-mutated (BRCAm) advanced ovarian cancer [47]. Olaparib belongs to the *N*-acyl piperazines class of compounds. The action of olaparib relies on the poly(ADP-ribose) polymerase PARP inhibition [48]. Poly(ADP-ribose) polymerases (PARPs) are a family of enzymes that catalyze the addition of poly(ADP-ribose) subunits onto themselves and other acceptor proteins and are involved in DNA repair [49]. The IC_50_ value towards PARP of olaparib is 6 nM [50]

Several synthetic strategies have appeared for the preparation of olaparib. Among these, the strategy disclosed by Scinopharm Taiwan [51] will be detailed. The authors of [51] state that the process was improved in terms of yield.

**Scheme 10:**
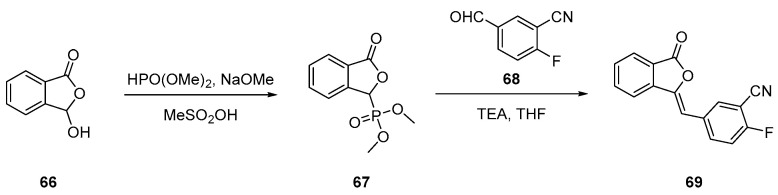
Preparation of intermediate **69** [51].

Nitrile derivate **69** (Scheme 10) is a key intermediate to form the phthalazine-one, from which two different pathways are built to the final product, olaparib.

Compound **69** is obtained by first reacting benzofuran **66** with dimethyl phosphonate to yield derivative **67**. This was used in the subsequent Horner–Wadsworth–Emmons (HWE) reaction with aldehyde **68** to yield alkene **69**.

**Scheme 11:**
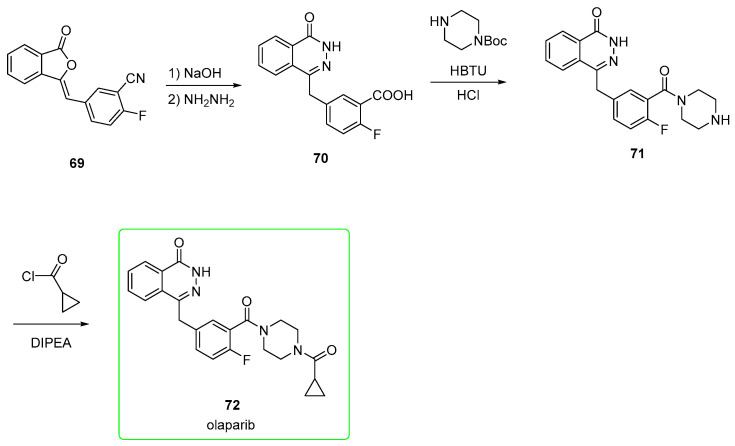
Preparation of olaparib (**72**) from synthon **69** [52].

In the first strategy (Scheme 11) [52], nitrile **69** is converted into the corresponding carboxylic acid by alkaline hydrolysis, and the phthalazine-one is formed upon the addition of hydrazine (compound **70**). This is subjected to condensation mediated by (2-(1H-benzotriazol-1-yl)-1,1,3,3-tetramethyluronium hexafluorophosphate (HBTU) followed by removal of the BOC group upon addition of HCl to provide the piperazine-containing amide **71**. This was eventually treated with cyclopropanecarbonyl chloride and DIPEA as a base to afford olaparib (**72**).

**Scheme 12:**
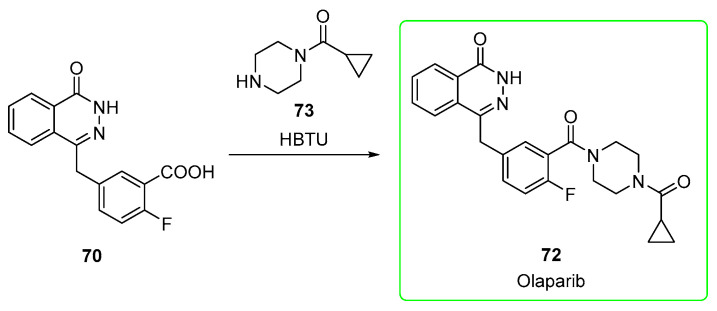
Preparation of olaparib (**72**) from synthon **69** [53].

In the second strategy (Scheme 12) [53], the carboxylic acid **70**, previously obtained from **69**, is treated directly with cyclopropyl(piperazin-1-yl)methanone **73** and HBTU to afford olaparib (**72**).

### 2.4. Ruxolitinib (Brand Name Jakafi^®^)

Ruxolitinib (**73**) is an orally administered, first-in-class Janus Kinase (JAK) 1 and 2 inhibitor that was recently approved for the treatment of patients with polycythemia vera (PV) [54], which is a rare slow-growing blood cancer characterized by increased erythrocyte production with a poorly understood etiology; IC_50_ values towards JAK1 and JAK2 are of 2.7, 4.5 nM, respectively [55].

In 2019, the FDA approved ruxolitinib for steroid-refractory acute graft-versus-host disease (SR-aGVHD) [56].

**Scheme 13:**
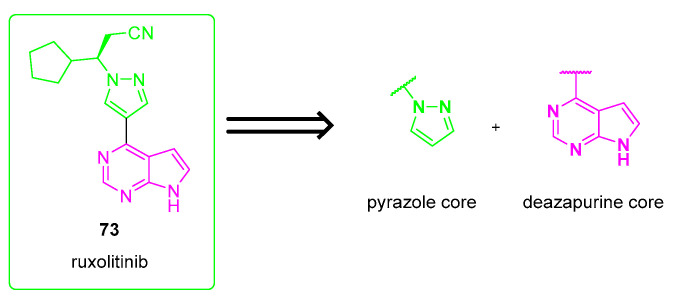
Retrosynthetic strategy for the preparation of ruxolitinib (**73**).

Ruxolitinib is a small molecule composed of a substituted pyrazole linked to a deazapurine core. The retrosynthetic strategy for the preparation of ruxolitinib suggests that the most straightforward and intuitive strategy is linking the deazapurine core with the *N*-substituted pyrazole (Scheme 13) through a Suzuki reaction [57].

**Scheme 14:**
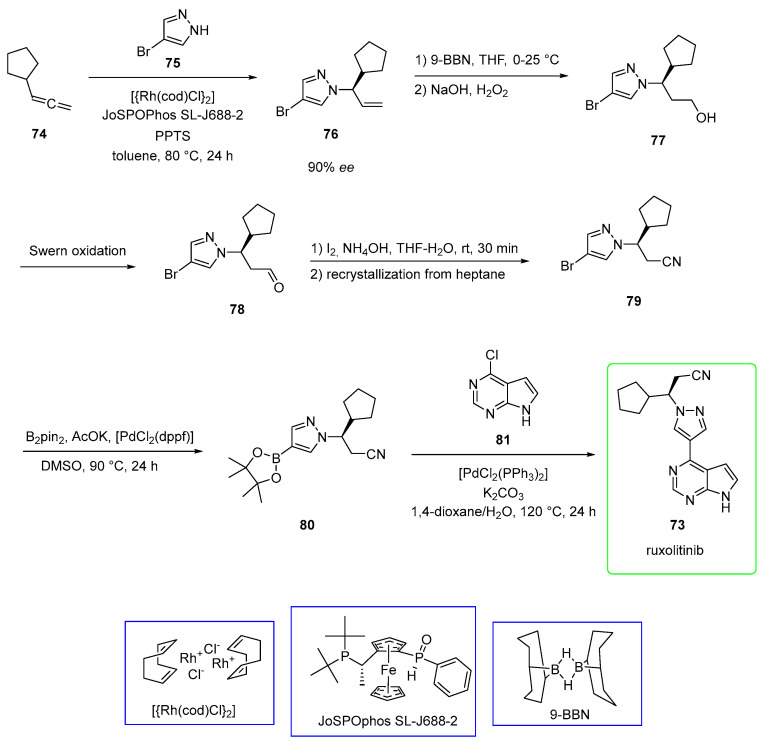
Preparation of ruxolitinib (**73**) via rhodium-catalyzed regioselective synthesis of pyrazole. Structures of [{Rh(cod)Cl}_2_], JoSPOPhos SL-J688-2, and 9-BBN are reported in blue boxes.

Haydl and co-workers, as an example, used a rhodium-catalyzed regioselective synthesis of pyrazole, which is the crucial synthon for the preparation of ruxolitinib (Scheme 14) [58]. Pyrazole **75** was added to terminal allene **74** to yield enantioenriched allylic pyrazole 76 (90% *ee*). The reaction was catalyzed by rhodium with ligand JoSPOPhos. The additional presence of pyridinium *p*-toluenesulfonate (PPTS, 0.2 equiv) played a significant role in obtaining the desired *N*_1_ product selectively. The subsequent hydroboration (9-borabicyclo[3.3.1]nonane or 9-BBN) followed by oxidation (H_2_O_2_) provided the primary alcohol **77**. Swern oxidation of **77** afforded aldehyde **78**, in turn, converted into nitrile **79** by the addition of ammonium hydroxide and iodine and purification by recrystallization from heptane. The installation of boron in the place of the bromide group was mediated by bis(pinacolato)diboron (B_2_pin_2_) and [1,1′-Bis(diphenylphosphino)ferrocene]palladium(II) dichloride ([PdCl_2_(dppf)] to yield boronic ester **80**. Eventually, Suzuki coupling [catalyzed by bis(triphenylphosphine)palladium chloride] of **80** with chloro-azapurine **81** provided ruxolitinib (**73**). The yield of the final product (over two steps) was 81%.

**Scheme 15:**
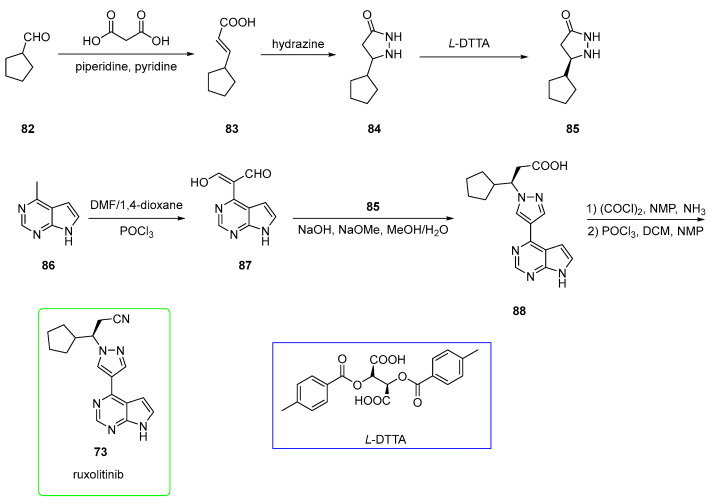
Preparation of ruxolitinib (**73**) suitable for industrial production. Di-*p*-toluoyl-*L*-tartaric acid (*L*-DTTA) is shown in the blue box [60].

Despite the elegant strategy, the reactions performed to synthesize the pyrazole moiety required high amounts of expensive and complex chiral catalysts [59]. Therefore, a more recent patent (see Scheme 15) [60] overcame these issues and made the process more scalable. The selected starting material was cyclopentanecarbaldehyde **82**, which was converted to €-alkene **83** in a Knoevenagel reaction involving malonic acid and piperidine/pyridine as a base. This was treated with hydrazine to afford cyclopentyl-pyrazolidinone **84**, which was resolved with di-*p*-toluoyl-*L*-tartaric acid (*L*-DTTA) to yield pure enantiomer **85**. On the other hand, the 6-methyl-7-deazapurine derivative **86** was treated with Vilsmeier reagent to yield aldehyde **87**. This was used with **85** in the presence of alkaline media to yield carboxylic acid precursor **88** of ruxolitinib. This was first treated with oxalyl chloride and ammonia to form the primary amide and later dehydrated to a nitrile upon using POCl_3_ to yield ruxolitinib. This route allowed the obtainment of **73** in a good yield, avoiding the use of complex and expensive catalysts.

### 2.5. Venetoclax (Brand Name Venclexta^®^)

Venetoclax (**103**) is an oral selective inhibitor of the prosurvival protein BCL-2 and, therefore, restores the apoptotic ability of malignant cells. It was developed by AbbVie. Venetoclax is approved in the USA for use as monotherapy in patients with chronic lymphocytic leukemia (CLL) [61]. Venetoclax addresses the same blood cancer subtype, chronic lymphocytic leukemia (CLL), as ibrutinib. Food increases venetoclax bioavailability by 3- to 5-fold, depending on the fat content, but no specific recommendation in terms of fat content in the meal is needed for the intake of venetoclax [62].

Recently, an acceptable yield and high-quality pathway with a desired solid polymorph property were published. This pathway also avoids the formation of undesired impurities [63,64].

**Scheme 16:**
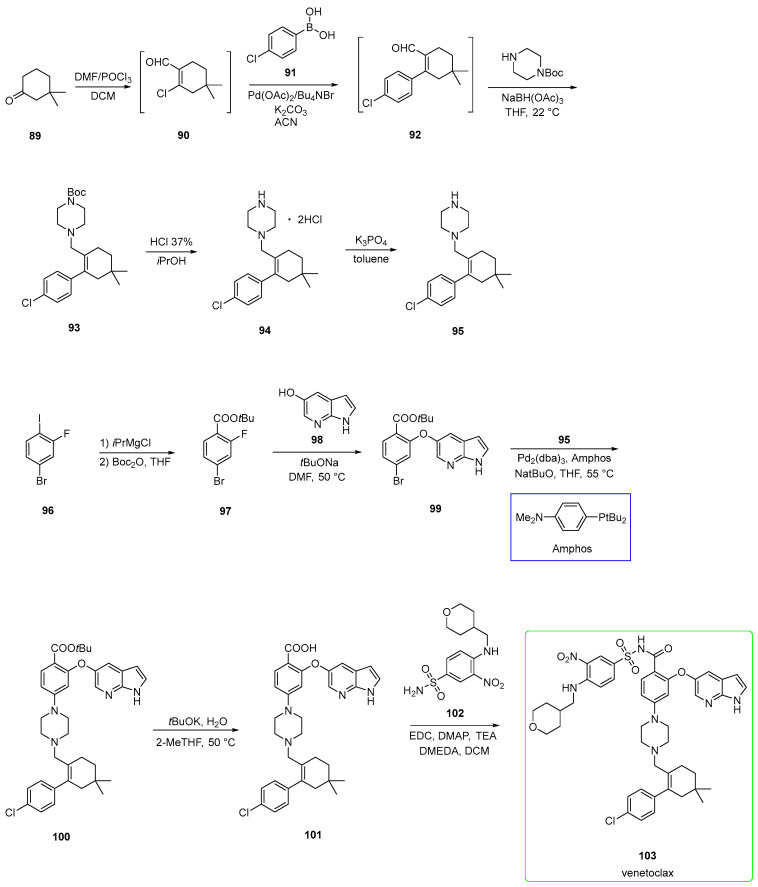
Preparation of venetoclax (**103**) suitable for industrial production [61,63]. Amphos is shown in the blue box.

The synthesis (Scheme 16) starts from 3,3-dimethylcyclohexan-1-one **89**, which was treated with Vilsmeier reagent to afford chlorinated aldehyde **90**. This was used in the next step without purification in the Suzuki-Miyaura coupling with boronic acid **91** to yield intermediate **92**. The subsequent reductive amination with *tert*-butyl piperazine-1-carboxylate and sodium triacetoxyborohydride afforded amine **93**. The BOC-protecting group was cleaved by 37% HCl in isopropanol to yield salt **94**, which was rendered a free base (**95**) upon treatment with K_3_PO_4_.

**Figure 2:**
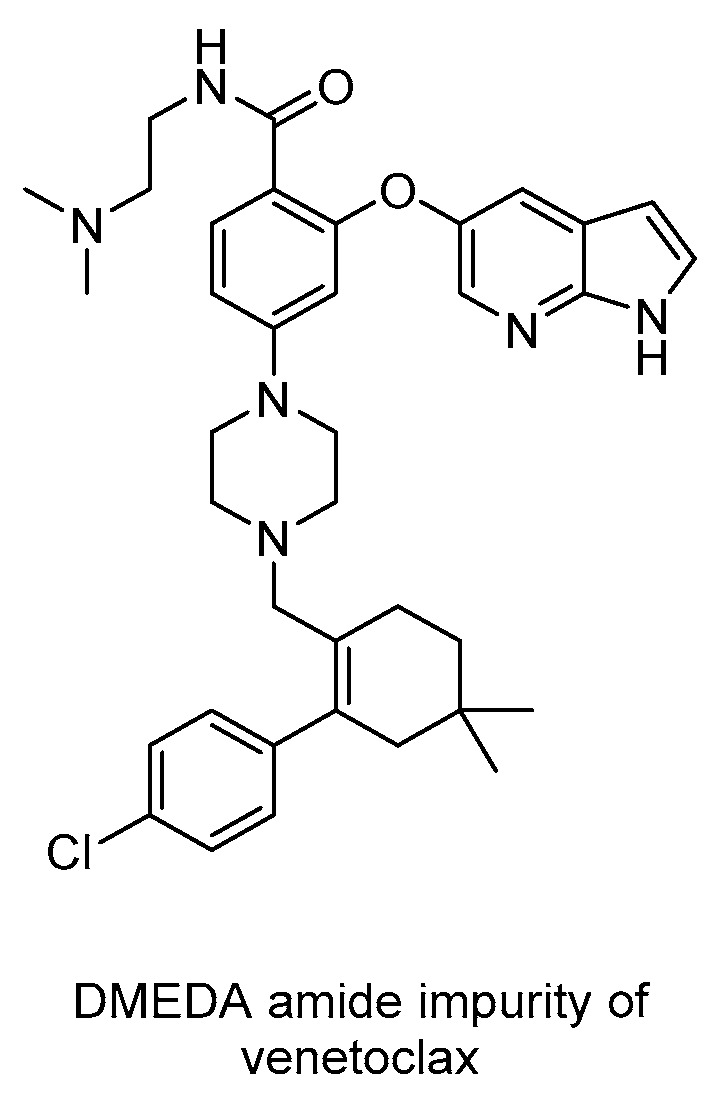
Structure of DMEDA amide impurity encountered in the synthesis of venetoclax (**103**).

On the other side, 4-bromo-2-fluoro-1-iodobenzene **96** was metalated with isopropylmagnesium chloride, followed by the addition of Boc_2_O to yield ester **97**. This underwent S_N_Ar with **98** in the presence of *t*BuONa as a base to yield diaryl ether **99**. This was subjected to Buchwald–Hartwig amination under Amphos and Pd_2_(dba)_3_ as a ligand and catalyst, respectively, to yield disubstituted piperazine **100**. This was hydrolyzed under alkaline conditions (*t*BuOK/H_2_O) to form **101**. The last stage involved the condensation between carboxylic acid and sulfonamide **102** under activation by EDC/DMAP and *N*,*N*-dimethylethylenediamine (DMEDA) to yield venetoclax. The last stage step was 84%. The presence of DMEDA led to the reformation of the desired product (from impurity) along with the corresponding DMEDA amide impurity (Figure 2) that was easily purified. This pathway, in summary, has led to a highly efficient and cost-effective synthesis.

### 2.6. Acalabrutinib (Brand Name Calquence^®^)

Developed by Acerta Pharma, acalabrutinib (**114**, Calquence^®^) is a Bruton’s tyrosine kinase inhibitor for the treatment of various hematological and solid malignancies [65].

In November 2019, the FDA approved acalabrutinib (owned by AstraZeneca) for adults with chronic lymphocytic leukemia (CLL) or small lymphocytic lymphoma (SLL) [66].

It is a selective, irreversible inhibitor (binding of acalabrutinib to the C481) of BTK that has improved pharmacologic features, including favorable plasma exposure, rapid oral absorption, a short half-life, and the absence of irreversible targeting to alternative kinases, such as an epidermal growth factor receptor (EGFR), tyrosine-protein kinase TEC, and IL-2-inducible tyrosine kinase (ITK) [67].

The half maximal inhibitory concentration (IC_50_) for BTK is 5.1 nM for acalabrutinib. The inhibition is weaker than that exhibited by ibrutinib [68].

Chemically, it contains an electron-deficient alkyne (responsible for irreversible inhibition) and the central imidazo-pyrazine.

**Scheme 17:**
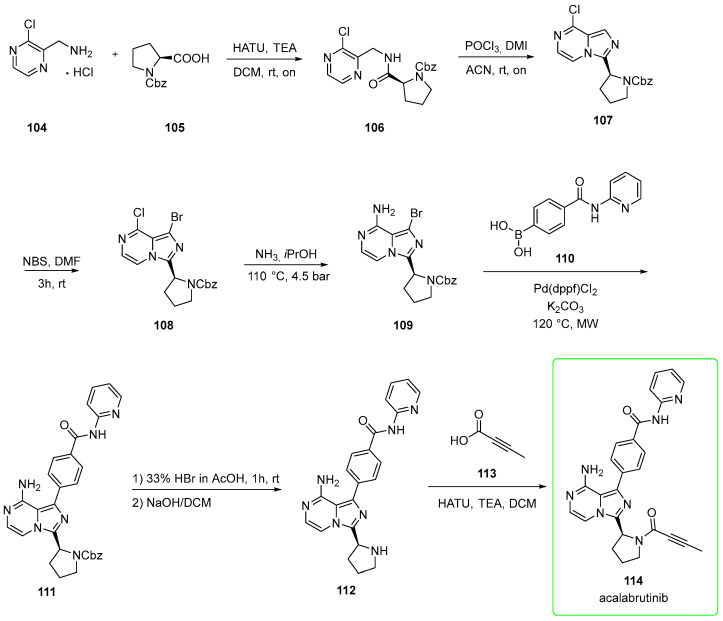
Preparation of acalabrutinib (**114**) [69,70].

Its synthesis (Scheme 17) [69,70] starts from (3-chloropyrazin-2-yl)methanamine **104** that is condensed with CBZ-*L*-proline 105 in the presence of HATU to afford the amide **106**. This was intra-molecularly cyclized under POCl_3_/1,3-dimethyl-2-imidazolidinone (DMI) conditions in acetonitrile to afford chloro-imidazopyrazine **107**. This was subjected to bromination (*N*-bromosuccinimide, NBS) on the imidazole scaffold to provide bromo derivative **108**. Treatment of **108** with ammonia in isopropanol afforded amine **109**. This was used in the subsequent microwave-assisted Suzuki-Miyaura coupling with boronic acid **110** using Pd(dppf)Cl_2_ as a catalyst to yield intermediate **111**. The deprotection of CBZ-protection mediated by HBr in acetic acid followed by neutralization in NaOH afforded amine **112**. Finally, **112** was condensed with 2-butynoic acid **113** in the presence of HATU to yield acalabrutinib (**114**).

The reported yield for this synthetic pathway was low and showed certain issues. For example, the process needed chromatography for the purification of the final stage and employed the corrosive hydrobromic acid in the deprotection stage. To overcome such a burden, more efficient pathways (cost-efficient and scalable) have been proposed.

**Scheme 18:**
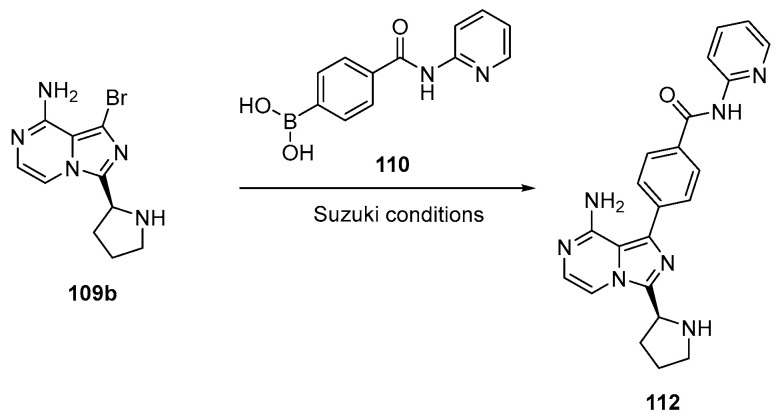
Preparation of intermediate **112**. This method avoids the deprotection stage, thus avoiding use of corrosive HBr [69].

For example, it has been reported that the product can be obtained via the unprotected pyrrolidine moiety (**109b**, Scheme 18), which can be easily purified by industrially viable purification methods, avoiding tedious chromatographic purification [69].

In this way, the deprotection of the CBZ group is not necessary, thereby providing an improvement to the preparation seen in Scheme 18. The thus-obtained precursor **112** can be converted into the product acalabrutinib (**114**) in the same way seen in Scheme 17, but the authors pointed out that acalabrutinib may be isolated from the reaction mixture by conventional methods, such as, but not limited to, filtration and/or centrifugation.

### 2.7. Tafamidis (Brand Name Vyndaqel^®^)

Tafamidis (**117**) is a small inhibitor discovered and developed by Scripps Research Centre in association with Pfizer in the treatment of transthyretin familial amyloid polyneuropathies (TTR-FAP). Recently, the FDA approved tafamidis for the treatment of heart disease (cardiomyopathy) that is caused by transthyretin-mediated amyloidosis (ATTR-CM) in adults. ATTR is caused by an abnormal deposit of specific proteins known as amyloids in the body’s organs and tissues [71].

**Scheme 19:**
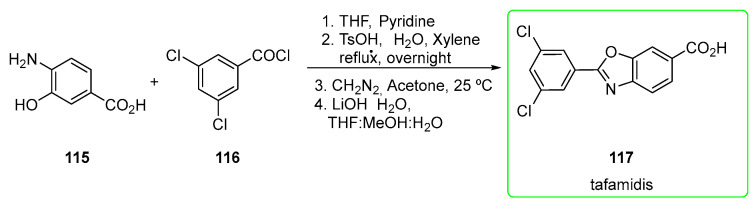
Preparation of tafamidis (**117**) [72].

Its main chemical feature is certainly a relatively low MW (MW = 308) and the easy synthetic pathway (Scheme 19) [72] to obtain it.

4-amino-3-hydroxybenzoic acid **115** was mixed with 3,5-dichlorobenzoyl chloride **116** in the presence of pyridine to yield the acylated form of **115**. The subsequent ring closure is mediated by *p*-toluenesulfonic acid monohydrate (TsOH-H_2_O), followed by esterification with diazomethane and final hydrolysis afforded tafamidis (**117**). Before hydrolysis, chromatography was used to purify the ester.

**Scheme 20:**
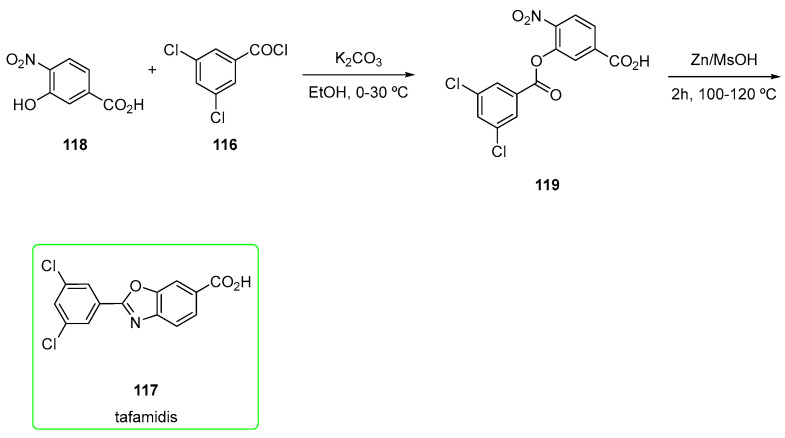
Alternative preparation of Zn-mediated tafamidis (**117**) [71].

**Figure 3:**
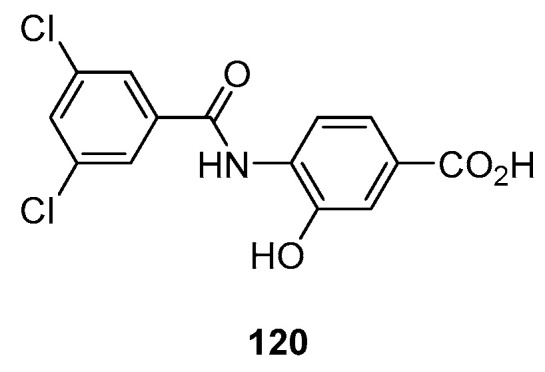
Structure of intermediate obtained by using Zn/acetic acid.

Another interesting approach (Scheme 20) involved the use of a nitro intermediate **118**. This was treated with **116** in the presence of potassium carbonate, and the corresponding ester was treated with zinc/methanesulfonic acid (MsOH). The use of a simple zinc-MsOH as a catalyst renders the protocol suitable for large-scale synthesis, providing a valuable synthetic tool for industrial application reductive cyclization reaction. The stoichiometry of the reaction was also very important: the main product benzoxazole-based **117** was obtained when using Zn (5 moles) and MsOH (15 times) at 100–110 °C. Interestingly, when using acetic acid (60 °C) instead of MsOH, the *N*-acylated corresponding intermediate **120** was obtained (Figure 3) [71].

## 3. Concluding Remarks

In this review, the top-selling orphan drugs (forecast for the year 2026) are discussed. The biological activity and, in particular, the synthesis of each derivative are detailed.

When possible, more than a single synthetic pathway was discussed: more efficient and scalable processes were discussed together with small scale-driven oriented ones. The majority (five out of seven) of the presented drugs belong to the oncology therapeutic category. The remaining two target diseases in the respiratory (elexacaftor/tezacaftor/ivacaftor) and central nervous system (tafamidis) disease categories.

The pyrazolo-pyrimidine nucleus, present in ibrutinib, and imidazo-pyrazine, present in acalabrutinib, represent well-studied scaffolds investigated by academia and pharmaceutical companies alike. The easy accessibility to these scaffolds and their well-explored structure–activity relationship (SAR) represent advantages from a synthetic point of view. These two drugs also share the presence of a Michael acceptor (acrylamide and alkyne, respectively) that makes them irreversible inhibitors, characterized by a Michael acceptor moiety able to form a covalent bond with the conserved Cys481 residue in the ATP binding site. Novel covalent warheads and a fine-tuning of their chemical neighborhood could allow for targeting specific amino acid residues and improve toxicity issues due to off-target binding. BTK inhibitors, such as ibrutinib and acalabrutinib, possess higher efficacy in patients with a high risk of disease and offer better tolerability in elderly and fragile patients [73].

The pyrrolo-pyrimidine scaffold of ruxolitinib also shares a similar central core, related to ibrutinib and acalbrutinib, with the difference of being a deazapurine.

Bruton tyrosine kinase and Janus Kinase (Jak) inhibitors (ruxolitinib) have seen a very prolific yield in terms of drug approval. These two targets offer a very promising opportunity in terms of reactivity and selectivity.

The triple-combination elexacaftor-tezacaftor-ivacaftor offers a nice example of how a synergistic effect brings an advantage in terms of drug efficacy and safety. Correctors of protein folding and/or potentiators act together, increasing CFTR channel function. Olaparib for BRCAm advanced ovarian cancer possesses a *N*-acylpiperazine moiety and a relatively easy synthetic preparation.

Venetoclax shares the piperazine moiety revealing that this aza-heterocycle is a very important synthon for many drugs.

In certain leukemias and certain solid tumors, apoptosis is a prominent (if not exclusive) mechanism associated with the induction of tumor remission. In addition, the expression of apoptotic modulators within a tumor appears to correlate with the sensitivity to traditional cancer therapies [74].

Therefore, molecules such as olaparib and venetoclax are of great importance in cancer therapy.

Benzoxazole tafamidis possess a particularly small size and a very easily accessible chemistry.

New tools and drug discovery approaches, from gene editing to artificial intelligence (AI)-powered screening and drug design, will provide the possibility for getting more and more orphan drug candidates in the future. With the implementation of AI in the manufacturing of pharmaceutical products, personalized therapies with precise doses, release parameters, and other required aspects can be manufactured according to individual patient needs [75]. As an example, the British company Benevolent Bio used a technological platform to screen out 5 compounds from 100 potential compounds that can treat ALS and confirmed that 4 of them were effective in curing motor neurodegeneration [76].

The benefits of orphan drug designation (for example, 7-year market exclusivity and up to a 50% tax credit for clinical trials that qualify) are encouraging pharma companies to invest in treating rare diseases. In addition, many rare diseases are still underexplored and could open the discovery of many other compounds in the next future.

## Data Availability

Not applicable.
